# Editorial: Mycobacteria-host interactions: genetics, immunity, pathology volume II

**DOI:** 10.3389/fcimb.2023.1216183

**Published:** 2023-05-18

**Authors:** Alexander S. Apt, Andrea Cooper, David N. McMurray

**Affiliations:** ^1^ Department of Immunology, Central Tuberculosis Research Institute, Moscow, Russia; ^2^ Department of Respiratory Sciences, University of Leicester, Leicester, United Kingdom; ^3^ Texas A&M University, College Station, TX, United States

**Keywords:** mycobacteria, immune response, gene expression, tuberculosis, COVID-19

Despite decades of research by some of the brightest scientific minds, and its designation by the World Health Organization as a global public health emergency, tuberculosis (TB) continues to be a major cause of disease and death world-wide. New vaccines, drugs and diagnostic tests will be needed before this ancient scourge can be brought under control. The development of those essential new tools will depend, in turn, on a better understanding of the interaction between the pathogen and the human host. The six articles that comprise this volume of our Research Topic make valuable contributions toward that improved understanding. These articles include a methods paper, three research articles, a review, and a perspective paper.

The paper by Kilinc et al. describes the development of a human cell-based *in vitro* infection model. With it, the authors examine the intracellular survival of *Mycobacterium avium*, an important cause of disease in immunocompromised patients characterized by drug resistance, prolonged therapy, and poor clinical outcomes. The authors optimized infection conditions for human monocyte/macrophages and the MelJuSo cell line. They used the BACTEC MGIT 960 to quantify intracellular bacterial survival, an assay that correlated with the colony-forming unit approach, but with less variation. They demonstrated that antimicrobial agents killed extracellular, but not intracellular, mycobacteria.

Mucosal-associated invariant T (MAIT) cells are CD8+ T cells that are activated by non-peptide small metabolites (e.g., riboflavin) and make proinflammatory cytokines and cytolytic molecules. To understand the mechanisms by which MAIT cells might protect against TB, Sharma et al. compared the transcriptome of MAIT cells activated with either *Escherichia coli* or *M. bovis* BCG. They found that MAIT cells activated by BCG had transcription signatures characterized by pro-survival, memory, and cytolysis (e.g., granulysin, perforin, TNFα) genes compared to cells stimulated with *E. coli*. The authors conclude that this transcription signature suggests an important role in mycobacterial resistance. Experiments with virulent mycobacteria, however, remain to be performed to more directly link this conclusion with *bona fide* TB infection.

The T-SPOT assay can be used to quantify TB-specific T cells in human blood that make IFNγ. Rao et al. hypothesized that platelet contamination in human peripheral blood mononuclear cells (PBMC) might be responsible for false-negative T-SPOT assays. Using PBMC derived from TB patients and healthy controls in Jiangxi, China, the authors modified the levels of platelet contamination and showed that increased levels were associated with inhibition of the T-SPOT assay. The authors attributed this effect to one or more soluble products of activated platelets, the identity of which remains to be elucidated.

In the third research article, Zhang et al. examined the association between single nucleotide polymorphisms (SNPs) in two genes (ALKBH5 and FTO) in pulmonary TB patients and healthy controls in Hefei, China. They observed that the expression of both genes was decreased in TB patients. Several SNPs in both genes were associated with clinical features of TB, such as drug resistance, hypoproteinemia, and leukopenia. The authors speculated that these SNPs may have some prognostic value in pulmonary TB. Validation of this conclusion in independent human population(s) is desirable.

Dendritic cells (DC) play multiple roles in the innate and adaptive immune responses to *M. tuberculosis*. They are essential for the activation of antigen-specific T cells in the lymph nodes draining the infectious focus in the lung. In an excellent review, Kim et al. examine the various subsets of DC and discuss their contributions to resistance to TB. A better understanding of the mechanisms by which DC may be alternatively activated or suppressed may allow the development of vaccination strategies that enhance resistance and/or host-directed therapies that improve antibiotic treatment outcomes.

The final article in this Special Issue is a perspective written by a well-established TB scientist, Ulrichs. The Covid pandemic had a major negative impact on TB disease control world-wide. The pandemic diverted resources in high-burden settings away from TB, resulting in a dramatic decline in the TB notification rates in many countries from 2020-2021. As a result, significant numbers of TB cases went undiagnosed and untreated, leading to increased transmission and a significant rise in TB mortality rates. In addition, lockdown measures driven by the Covid pandemic prevented many patients from accessing their anti-TB drugs, leading to poor treatment outcomes, and likely increasing drug resistance. In addition, there is some evidence that the two infections synergize to make both worse in co-infected individuals.

Taken together, these six articles address diverse aspects of the host-pathogen interaction in mycobacterial infections and make important contributions to the development of improved methodologies for studying these diseases, both *in vitro* and *in vivo*.

## Author contributions

All authors listed have made a substantial, direct, and intellectual contribution to the work and approved it for publication.

